# Clinical characteristics and genetic analysis of A20 haploinsufficiency

**DOI:** 10.1186/s12969-021-00558-6

**Published:** 2021-05-24

**Authors:** Dan Zhang, Gaixiu Su, Zhixuan Zhou, Jianming Lai

**Affiliations:** Capital Institute of Pediatrics, 2 yabao road, Chaoyang District, Beijing, China

**Keywords:** A20 haploinsufficiency, Behçet-like syndrome, Tumor necrosis factor antagonists

## Abstract

**Purpose:**

To evaluate the clinical and genetic characteristics of 3 children with Haploinsufficiency of A20 (HA20). Methods:The clinical and genetic testing data of 3 children with HA20 treated at Capital Institute of Pediatrics (CIP) between August 2016 and October 2019 were retrospectively analysed.

**Result:**

Patient 1 presented with arthritis and inflammatory bowel disease, patient 2 presented with axial spinal arthritis and lupus-like syndrome, and patient 3 presented with recurrent oral ulcers, gastrointestinal ulcers, and perianal abscesses. Regarding laboratory tests, patients were found to have elevated white blood cell (WBC) count, C-reactive protein (CRP) and erythrocyte sedimentation rate (ESR). The CRP and ESR was reported to be high in all the patients. The WBC was reported to be high in patient 1 and 3. Patient 2 was positive for antinuclear antibodies, anti-Sjögren’s syndrome antigen A, dsDNA, rheumatoid factor and Coombs test. Genetic testing showed that all three patients had heterozygous mutation in TNFAIP3 gene. As for the treatment, patient 1 was treated with TNFα antagonist, patient 2 was treated with TNF α antagonist and sulfasalazine, and patient 3 was treated with corticosteroids and thalidomide. Patients 1 and 2 were followed for four and 3 months, respectively. There was an improvement in joint and gastrointestinal symptoms; inflammatory indices and rheumatoid factor (RF) were normal, and dsDNA and Coombs test became negative. Patient 3 was treated at another hospital and showed gradual improvement in oral ulcers and perianal abscesses.

**Conclusion:**

HA20 is a single-gene auto-inflammatory disease caused by mutation in tumour necrosis factor (TNF)-α-induced protein 3 (TNFAIP3) gene. It may present as Behçet-like syndrome and resemble various other autoimmune diseases as well. Corticosteroids and immunosuppressive agents are effective treatments, and cytokine antagonists can be used in refractory cases. Whole-exome genetic testing should be proactively performed for children with early-age onset or Behçet-like syndrome to achieve early diagnosis and accurate treatment.

## Key points

1. HA20 presents as Behçet-like syndrome and can reaemble various other autoimmune diseases as well.
2. Corticosteroids and immunosuppressive agents are effective treatments, and cytokine antagonists can be used in refractory cases.

## Background

A20 haploinsufficiency is an autosomal dominant hereditary disease caused by a pathological mutation in tumour necrosis factor (TNF)-α-induced protein 3 gene [1, 2]. As a result, production of nuclear factor (NF)-κB regulatory protein A20 encoded by TNFAIP3 gene is insufficient, and clinical signs resemble Behçet’s disease [3, 4]. However, these clinical signs appear earlier in HA20, and the age of disease onset is most likely before 10, including recurrent appearance of painful oral, genital, and/or gastrointestinal ulcers. With the extensive use of whole-exome genetic testing in recent years, reports of this disease have gradually increased. In most reports, the clinical presentation of this disease is primarily resembles Behçet-like disease, but few other complications, like other autoimmune diseases such as lupus-like syndrome and autoimmune cytopenia. Steriod and immunosuppressive agents are the primary treatments. TNF-α antagonists or other biologics has not been widely used. The three patients of HA20 recently diagnosed at our hospital had an early age onset. In addition to presentation with Behcet’s disease, one patient presented with lupus-like syndrome and one presented with Crohn’s disease. Two of three patients were treated with biologics, and showed significant clinical improvement and few side effects. The clinical and genetic testing results of all three patients were analysed in order to improve our understanding of diagnosis and treatment of HA20.

## Methods

Clinical data. The clinical characteristics of 3 children (1 male, 2 female) with HA20 who were admitted to and diagnosed at Affiliated Children’s Hospital of Capital Institute of Pediatrics between August 2016 and October 2019 were retrospectively analysed. Medical history was obtained, including age of onset, time to diagnosis, medical history, physical examination, laboratory tests, electronic endoscopy, and whole-exome genetic testing. Primary observational indices were as follows: (1) Clinical presentation: fever, oral ulcers, genital ulcers, abdominal pain/diarrhoea, skin lesions, eye lesions, arthritis, kidney involvement; (2) Laboratory tests: conventional blood tests, C-reactive protein (CRP), dynamic erythrocyte sedimentation, conventional urine tests, antinuclear antibody (ANA) panel; (3) Arthrography and electronic endoscopy; and (4) Whole-exome genetic testing. After obtaining consent from the child’s guardian, approximately 2 mL (EDTA anticoagulant) of peripheral blood was collected from each child and their parents, and genomic DNA was extracted using the QIAamp whole blood DNA extraction kit (Qiagen, Hilden, Germany). Exon capture kit (probe synthesised by Twist Bioscience, San Francisco, CA, USA) was used. The Illumina NovaSeq 6000 s-generation sequencer (San Diego, CA, USA) was used to capture and sequence the coding exon regions of the sample gene. BWA software was used to compare the sequences with a reference genome (version hg19), and analyse statistical data of sequencing depth, uniformity, probe specificity, and other data performed. GATK software was used to detect polymorphic sites from comparison data of each sample, and analyse single-nucleotide polymorphism (SNP) and indel mutation data. The SNPs and indels obtained from screening had a frequency of less than 0.05 in the 1000 Genomes Project, ESP6500si, ExAC_ALL, ExAC_EAS, and the myGenostics 1000 normal individual databases. In addition, the results predicted from SIFT, PolyPhen2, MutationTaster, GERP++, and other databases, which were pathogenic, loci served as disease-related candidate loci.

## Results

**Fig. 1:**
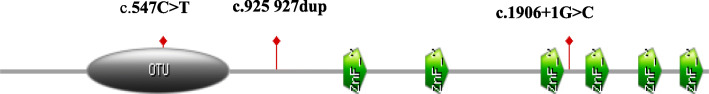
Structural domains of the TNFAIP3 protein

Table 1 summarizes the clinical presentations of the 3 patients. Table 2 summarizes the laboratory test data. Figure 1 shows the results of whole-exome genetic testing.

**Table 1:** Table 1

	Patient1	Patient2	Patient3
Fever	+	–	+
Oral ulcer	–	+	+
Genital ulcer	–	–	–
Gastrointestinal elcer	+	–	+
Rash	–	–	–
Oculopathy	–	–	–
Arthritis	+	+	–
Renal damage	–	+	–

**Table 2:** Table 2

	WBC (×10^9^/L)		CRP (mg/L)		AESR (mm/60 min)		PCT	Routine urine test/24 h proteinuria	ANA
	Prior treatment	Post treatment	Prior treatment	Post treatment	Prior treatment	Post treatment			
Patient1	34.06	7.3	150	2.7	47	6	< 0.5	–	–
Patient2	5.61	3.68	73	0.75	87	8	< 0.5	–	ANA 1:320 dsDNA positive
Patient3	17	N	17.5	N	31	N	< 0.5	–	–

The sites of gene mutation in TNFAIP3 in those 3 patients were c.1906 + 1G > C heterozygous mutation, c.925_927dup heterozygous mutation, and c.547C > T heterozygous mutation. All of those mutations do not appear in 1000 Genomes Project, ESP6500si, ExAC_ALL, ExAC_EAS, or myGenostics 1000 normal individual databases. Among these mutations, c.1906 + 1G > C and c.547C > T were predicted by GERP++ as loci in a conserved region. c.1906 + 1G > C and c.925_927dup were confirmed by Sanger sequencing as spontaneous mutations. The before c.547C > T mutation in one of the patients was confirmed by Sanger sequencing as being inherited from his mother.

As shown in Fig. 1, the c.547C > T locus falls within the ovarian tumour (OTU) domain of OTU-like cysteine proteases. This family consists of a group of predicted cysteine proteases homologous to OTU gene in Drosophila, which includes proteins from eukaryotes, viruses, and pathogenic bacteria. Conserved cysteine, histidine, and possibly aspartic acid residues represent catalytic residues in this putative protease family. These mutations may affect the function of protein domain. Among them, 1906 + 1G > C may affect the alternative splicing of exons, thereby affecting protein function.

TNFAIP3 gene has an autosomal dominant mode of inheritance. Based on these results, our 3 patients showed the possibility of pathogenicity at genetic level.

### Patient 1

**Fig. 2:**
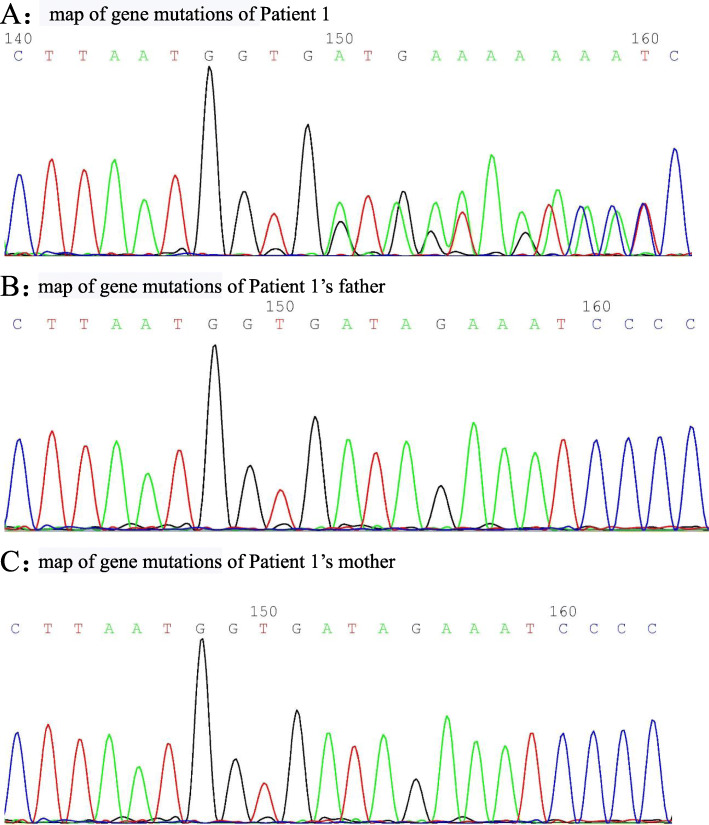
**a** Map of gene mutations of Patient 1. **b** Map of gene mutations of Patient 1’s father. **c** Map of gene mutations of Patient 1’s mother

A 3 years and 2 months girl was admitted with “swelling and pain in multiple joints for 6 months and intermittent fever with diarrhoea for over 4 months”. Six months before admission, the patient developed swelling and pain at her right ankle with limited mobility without any obvious cause. There was no fever, vomiting, abdominal pain, rash, eye discomfort. She received no treatment at that moment. Four months before admission, the patient developed intermittent fever accompanied by diarrhoea, but no abdominal pain or bloody stool. She continued to have joint swelling and pain. Conventional blood tests at local hospital showed that WBC was 15.8 × 10^9^/L, classified primarily as neutrophils, haemoglobin was 106 g/L, CRP was 42.7 mg/L, and dynamic erythrocyte sedimentation rate (AESR) was 50 mm/60 min. Magnetic resonance imaging (MRI) of the joints indicated synovial thickening, which was considered as “infectious arthritis”. However, There was no improvement with anti-infective treatment. Therefore, synovectomy and joint irrigation was performed. After the operation, the patient had intermittent fever with diarrhoea, bloody stool, bilateral knee swelling, pain, and limited mobility. Physical examination and treatment at our department revealed a 91 cm and 11.5 kg boy, with fever, flush, 4 cm swelling of fourth proximal interphalangeal joint at right hand with mild tenderness without limited mobility, 21 cm swelling of bilateral knees with positive floating patella test and limited flexion, swelling of right ankle without tenderness with plantar flexion of 5° and dorsal extension of 5°. After admission, conventional blood test showed that WBC was 34.06 × 10^9^/L, CRP was 150 mg/L, and AESR of 47 mm/60 min. Tests for tuberculosis, fungal infection, parasite infection, Epstein-Barr virus, and other pathogens were negative. The antinuclear antibody (ANA) panel was negative. Immunity tests showed IgA was less than 0.0647 g/L, and IgG and IgM were normal. Cellular immunity was normal. Enhanced computed tomography of the thorax and abdomen were negative. Because of fever and diarrhoea presented during the course of her disease, the height and weight of the patient were in the 3rd percentile for the same sex and age, suggesting growth delay. Multiple colorectal ulcers on endoscopic examination and a new c.925-927dup (p. Val309dup) mutation at TNFAIP3 gene on subsequent whole-exome genetic testing suggested a diagnosis of A20 haploinsufficiency (see Fig. 2 for a map of gene mutation). The patient received oral thalidomide and two doses of 6 mg/kg infliximab, and then switched to 20 mg adalimumab subcutaneously every 2 weeks because of allergy of infliximab. Currently, the patient has been treated with adalimumab for 3 months. Her fever and joint swelling and pain improved significantly. Her conventional blood tests and inflammatory indicators returned to normal, and endoscopic examination indicated ulcer had disappeared.

### Patient 2

**Fig. 3:**
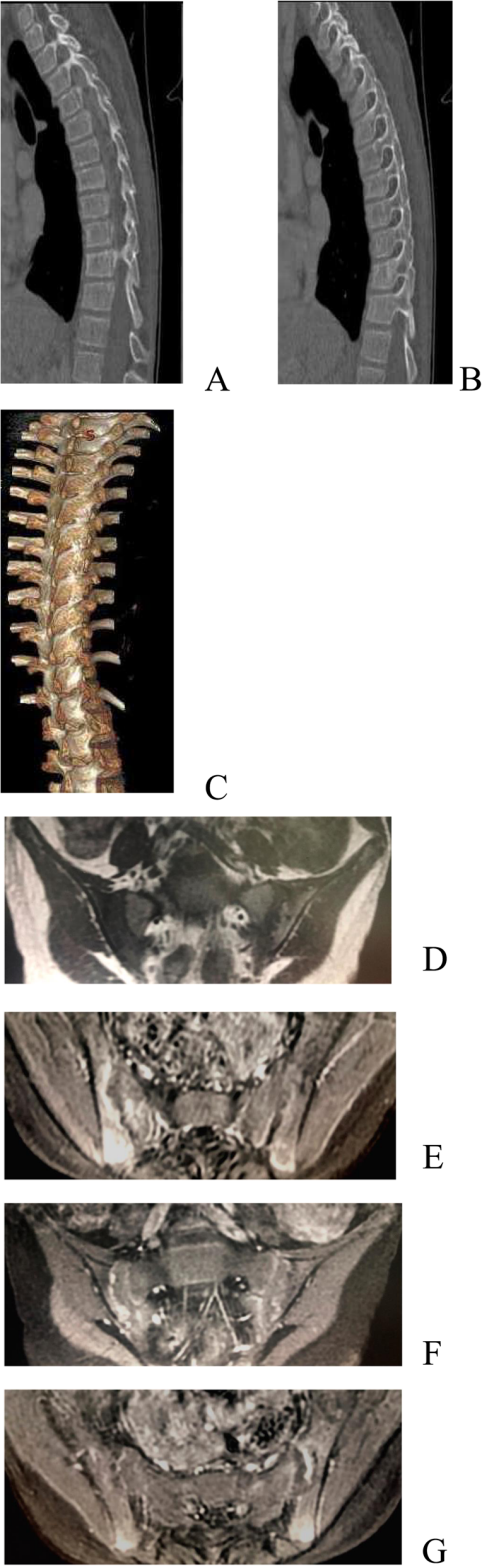
**a** Map of gene mutations of Patient 2. **b** Map of gene mutations of Patient 2’s father. **c** map of gene mutations of Patient 2’s mother

**Fig. 4:**
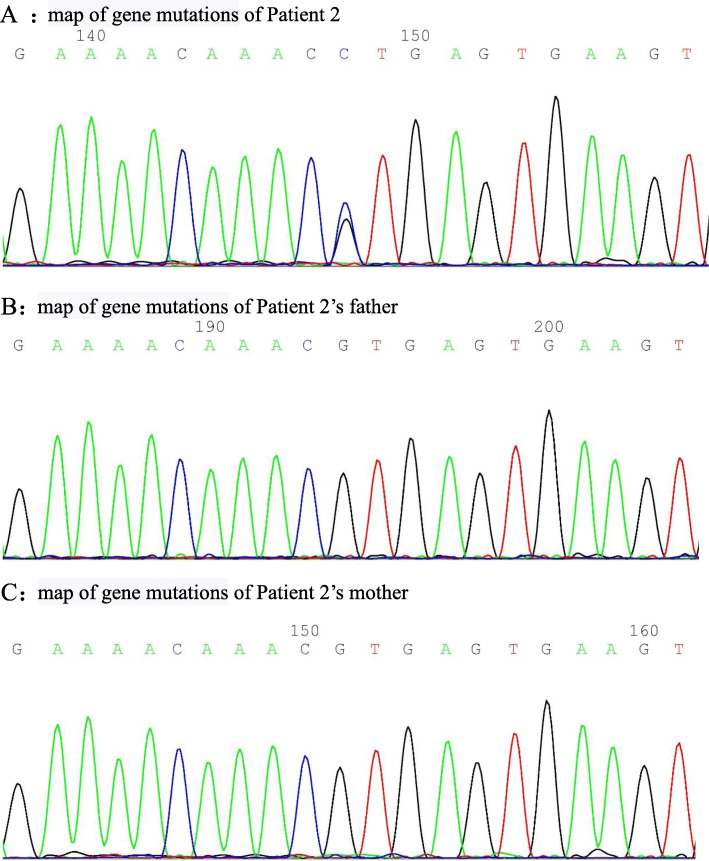
**a** Map of gene mutations of Patient 3. **b** Map of gene mutations of Patient 3’s father. **c** Map of gene mutations of Patient 3’s mother

An 11 years and 7 months girl was admitted with “recurrent lower back pain for over two years”. Over 2 years before admission, her patient discovered that the girl had thoracic kyphosis and recurrent oral ulcers. She had no special treatment. Later, the patient developed lower back pain and inability to bend down. There was no fever, vomiting, abdominal pain, rash, joint swelling, or eye discomfort. Rheumatoid factor (RF) examination at local hospital was 37.5 IU/mL, positivity for HLA-B27, normal WBC, CRP of 42.7 mg/L, and AESR of 50 mm/60 min,. Her physical examination at our department showed a 105 cm and 18.4 kg girl, with developmental delay, emaciation, scoliosis, thoracic kyphosis, positive spinous process tenderness, positive Schober’s test, and bilateral positive number 4 sign. Conventional blood tests showed normal WBC, Hb of 100 g/L, CRP of 73 mg/L, and AESR of 87 mm/60 min. The ANA panel showed a ratio of 1:100, anti-Sjögren’s syndrome antigen A (SSA)++, and RO 52+++. The Coombs test was positive, cellular and humoral immunity were generally normal. Conventional urine tests and 24-h urinary protein were normal. Complement was normal. Enhanced sacroiliac MRI indicated inflammatory changes. The patient had a history of recurrent oral ulcers and alopecia which became more and more obvious, and systemic lupus erythematosus was strongly suspected. Renal biopsy was not performed. Juvenile idiopathic arthritis was diagnosed, and the patient was administered oral sulfasalazine. Her low back pain gradually subsided after treatment. Eight months before admission, lower back pain came back, and examination showed an ANA of 1:1000, SSA+++, and positive double-stranded DNA (dsDNA; enzyme-linked immunosorbent assay). Enhanced sacroiliac MRI re-examination suggested exacerbation of the inflammatory changes. Together with her presentation of autoimmune haemolytic anaemia and arthritis, a diagnosis of systemic lupus erythematosus was considered in accordance with the 1997 ACR standard. Oral methylprednisolone, hydroxychloroquine, and sulfasalazine were administered for 6 months, then discontinued completely. Two months before admission, her lower back pain aggravated. Enhanced MRI of thoracic and lumbar spine at our hospital revealed extensive small pyramidal arthritic changes (Fig. 3). Enhanced MRI of sacroiliac joint revealed re-aggravation. The patient was treated with sulfasalazine and infliximab. Her joint symptoms subsided significantly. Since her height was only 130 cm at age 11, which is below the 3rd percentile for the same sex and age, she was diagnosed with dwarfism. Besides, her whole-exome genetic testing indicated a new mutation in TNFAIP3 at c.1906 + 1G > C (see Fig. 4 for a map of gene mutations). Therefore, she was diagnosed with A20 haploinsufficiency. Infliximab (6 mg/kg) was administered 3 times. At follow-up examination, the patient did not report back pain, and CRP and AESR were normal.

### Case 3

**Fig. 5:**
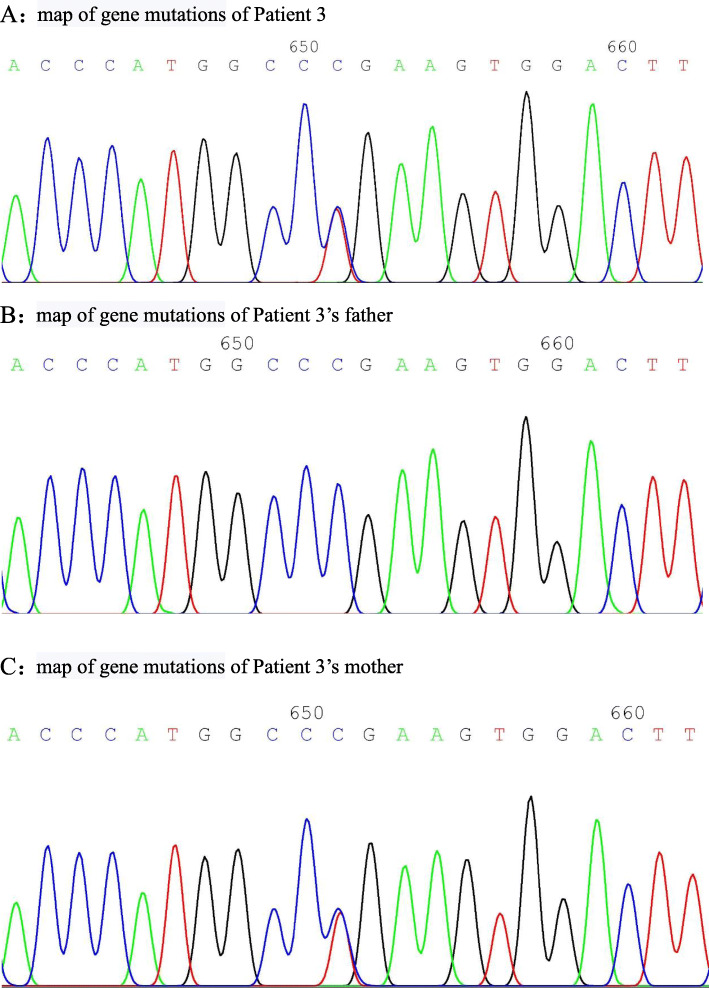
CT: **a, b**: reconstruction of thoracic CT sagittal bone window, **c**: three-dimensional image showing extensive osseointegration of thoracic spine and disappearance of vertebral facet joint space. MRI: **d**, T1WI sequence of the sacroiliac joint showing that the bilateral sacroiliac joint space is widened, and the zygomatic side articular surface is jagged. **e**, FSPD sequence showing multiple patchy high signals below the sacroiliac joint, suggesting bone marrow oedema with the right sacroiliac joint as the focus; **f**, **g**, T1WI sequence of enhanced fat suppression scans showing significant enhancement of the bilateral articular surfaces, indicating the presence of active inflammation

A 4 years and 5 months boy was admitted with “intermittent fever with perianal abscesses for over 3 years and intermittent fever for 2 years”. Three years before admission, the patient developed perianal abscesses and fever with no obvious cause. There was no cough, vomiting, abdominal pain, rash, joint swelling and pain, or eye discomfort. The patient was symptomatically treated with topical and intravenous antibiotics and intermittent steroids at local hospital, but the symptoms were recurrent after treatment. Two years before admission, the patient developed fever with no other symptoms. The patient was diagnosed with bacterial infection at local hospital and showed improvement after antibiotic and steroids. The fever recurred at an interval ranging from 3 days to 2 months. Six months before admission, he developed recurrent oral ulcers, with 4–5 episodes within 6 months. There was intermittent abdominal pain but no diarrhoea, no bloody stool, or other symptoms. Dynamic monitoring and conventional blood tests showed WBC of 17 × 10^9^/L, CRP of 105 mg/L, and AESR of 31 mm/60 min. The patient was diagnosed at our department with pre-existing pigment deposition in the perianal skin. The patient was negative for tuberculosis and fungal, parasitic, Epstein-Barr virus, and other infections. Antinuclear antibody and antineutrophil cytoplasmic antibody panels were negative. Neutrophil phagocytosis was normal. Whole-exome genetic testing found a c.547C > T (p.Arg183Ter) mutation in TNFAIP3 (see Fig. 5 for the gene mutation map). The mutation originated from the mother, who also had a history of recurrent oral and genital ulcers for over 20 years. The father did not carry the mutation. Therefore a diagnosis of A20 haploinsufficiency was considered. Later, complete endoscopic examination was performed at local hospital, which indicated ileocecal ulcers. Hormone and thalidomide immunosuppressive therapy were given to the boy. There was occasional recurrence of periodic fever during steroid dose reduction. Conventional blood tests during the episodes showed elevated WBC, CRP, and AESR. Currently, hormones have been discontinued, and the disease course is relatively stable.

Figures 2, 4 and 5 are maps of gene mutation of 3 cases.

## Discussion

A20 haploinsufficiency (HA20) is an autosomal dominant genetic disease caused by mutation of TNF-α-induced protein 3 (TNFAIP3) gene [5–7]. The cause of this disease is insufficient production of A20 resulting in negative feedback inhibition of NF-kB signalling pathway. it presents with the characteristics of autoinflammatory disease. Laboratory examination of this disease may reveal elevated CRP and AESR at disease onset as well as the presence of autoantibodies to varying degrees, thus patients may present with characteristics of autoimmune disease. Colchicine treatment is effective in some patients, whereas immunosuppressive agents (including systemic glucocorticoids, slow-acting antirheumatic drugs, and cytokine inhibitors) alone or combination therapy are used in other patients.

NF-κB is a family of dimeric proteins composed of Rel domains. When stimulated by various inflammatory factors (such as viruses, TNF, B-cell activating factor, lymphotoxin, and others), NF-κB can induce the expression of various genes, resulting in the production of cytokines that participate in inflammatory response. These cytokines are associated with inflammation, including chemokines, adhesion factors, and enzymes that produce secondary inflammatory mediators (such as cyclooxygenase and inducible nitric oxide synthase) and promote the inflammatory response process. In addition, some proteins induced by NF-κB such as TNF-α can act on NF-κB via negative feedback, therefore resulting in continuous production of inflammatory factors and aggravating the inflammatory response. Continuous activation of NF-κB signalling pathway and persistent inflammatory response can cause pathological changes in the body, such as systemic lupus erythematosus, rheumatoid arthritis, cancer, and other diseases associated with inflammation.

The A20 protein, encoded by TNFAIP3 gene and known as TNF-α-inducing protein 3, was first discovered in 1990 by Dixit et al. in human umbilical vein endothelial cells treated with TNF-α [8, 9]. Later, A20 was found to be expressed in epithelial cells, dendritic cells, and T and B lymphocytes. A20 protein is a negative regulator of the NF-κB signalling pathway and plays a fundamental role in a variety of physiological and pathological processes such as immunity, apoptosis, inflammation, and cancer [10]. It regulates the immune response by preventing NF-κB from overstimulation by various exogenous stimuli. The A20 protein consists of an N-terminal OTU structural domain and a C-terminal OTU domain containing 7 zinc finger domains. The deubiquitinating enzyme activity of the OTU domain can cause the receptor-interacting protein in the NF-KB signalling pathway to undergo deubiquitination [11], inhibiting its degradation by proteases. The ubiquitinase enzyme activity of the repetitive zinc finger structure can cause ubiquitination and degradation of Lye8 in receptor-interacting protein. The deubiquitinating enzyme structural domain of A20 can antagonize ubiquitination upstream of a necessary regulator of NF-κB (NEMO) by activating various mechanisms; this regulator is a regulatory subunit of the IKK kinase complex. The IKK complex has two catalytic subunits (IKKa and IKKb) and a subunit that regulates the NF-κB pathway (NEMO/IKKg). IKK regulates non-canonical pathways through IKKa. A20 ubiquitination of NEMO can down-regulate IKK activation by blocking IKK phosphorylation. IKK activation inhibits the phosphorylation of IKKa protein, resulting in its degradation and dissociates from NF-κB, and then translocation to the target gene for transcription. Therefore, loss of A20 leads to NF-κB pathway activation. Mutations in either the N- or C-terminus of TNFAIP3 gene result in loss of A20 function.

HA20 is a novel single-gene auto-inflammatory disease first reported in 2016 by Zhou et al. [12], who found that its primary phenotype is Behçet’s disease-like symptoms [13], including recurrent oral ulcers, genital ulcers, and gastrointestinal symptoms [14]. However, numerous studies have shown that some patients exhibit not only the characteristics of auto-inflammatory diseases, but also those of various autoimmune diseases. In genome-wide association studies, TNFAIP3 polymorphisms were found to be associated with a variety of autoimmune [15] and autoinflammatory diseases [16], such as systemic lupus erythematosus [17], Sjögren’s syndrome, Crohn’s disease, rheumatoid arthritis, Still’s disease in adults, juvenile idiopathic arthritis, psoriatic arthritis, Hashimoto’s thyroiditis [18], autoimmune lymphoproliferative syndrome [19], type 1 diabetes, and psoriasis. To date, 24 pathogenic variants of A20 have been reported. Significant clinical heterogeneity exists even in patients with the same variant of TNFAIP3 [20]. Therefore, HA20 may have unexpected clinical phenotypes. No cases of HA20 have been reported in China. A search of PubMed database with terms “A20 haploinsufficiency” and “TNFAIP3” (as of October 2019) yielded a total of 24 relevant articles involving 35 patients in 14 families, of which 12 patients were male and 23 patients were female; the male/female ratio was 1:2 and the average age of onset was 6.3 years. Of the 35 patients, 18 had fever, 17 had oral ulcers, 18 had genital ulcers, 5 had lymphadenitis, 12 had gastrointestinal symptoms such as abdominal pain and diarrhoea, 5 had gastrointestinal ulcers, 2 had perianal abscesses, 15 had arthritis, 8 had skin lesions (of which there was 1 case of vitiligo), 7 had Hashimoto’s thyroiditis, and 3 had renal involvement (haematuria, proteinuria). There was only 1 case of ocular lesions, 1 case of malignant cancer, 1 case of deep vein thrombosis with arterial inflammation, and 1 case of Raynaud’s phenomenon. One case presented with systemic juvenile idiopathic arthritis with macrophage activation syndrome and interstitial pneumonia (presenting as fever, abnormal liver function, hypertriglyceridemia, hypofibrinogenemia, and significant ferritin elevation). One case presented with juvenile systemic lupus erythematosus and lupus nephritis. In conventional blood tests, all 24 patients had elevated WBCs, CRP, and AESR, of which 2 were positive for ANAs, 1 was positive for SSA, 1 was positive for dsDNA, 1 had complement deficiency, 2 were positive for HLA-B27, and 1 had lupus nephritis revealed by renal biopsy, all of which were consistent with the characteristics of the HA20 clinical phenotype.

Mouse studies have shown that the disease phenotype may depend on the major cell types affected. Mice who are homozygous for A20 loss can develop early-onset auto-inflammatory diseases. Mice with bone marrow cells lacking A20 develop polyarthritis similar to rheumatoid arthritis in humans, whereas intestinal cell-specific A20 deficiency can result in intestinal inflammation in mice [21]. Cell-specific loss of A20 in B cells and dendritic cells also results in a corresponding clinical phenotype with the production of autoantibodies. In addition, somatic deletion and biallelic mutations of TNFAIP3 have been found in B-cell lymphoma, suggesting that A20 plays a role in suppressing cancer. Therefore, factors such as genetics and the environment may play a determinative role in the disease phenotype. In addition, the heterogeneity of HA20 also appeared in the same family, with clinical manifestations ranging from periodic fever-like disease and Behçet’s disease to rheumatoid arthritis.

In our 3 patients, patient 1 presented primarily with arthritis and inflammatory bowel disease, patient 2 presented primarily with severe spinal arthropathy and lupus-like syndrome, and patient 3 presented primarily with Behçet-like syndrome. Patients 1 and 3 had recurrent fever that tended to resolve on its own without any fixed cycle, and conventional blood tests during febrile periods showed elevated WBC and inflammation indicators, exhibiting the characteristics of auto-inflammatory diseases. Patient 2 had no fever or gastrointestinal symptoms but was positive for ANA, SSA, dsDNA, and Coombs test, and exhibited severe spinal arthritis and lupus-like syndrome, which are characteristic of autoimmune diseases. The 3 patient also showed diverse clinical phenotypes, in agreement with literature reports. The heterozygous mutation in patient 3 in this group originated from the patient’s mother, who had a history of recurrent oral and genital ulcers since childhood, consistent with the characteristics of onset of familial HA20. Both patients 1 and 2 had varying degrees of delayed growth, which was considered as associated with long-term inflammation and diarrhoea in patient 1 and long-term inflammation and limited growth due to severe spinal arthritic joint fusion in patient 2. Because patients with HA20 had such heterogeneous clinical presentations, genetic screening of patients based on clinical characteristics alone is unlikely to be sensitive enough to detect all relevant cases. Whole-exome/genomic sequencing is becoming more widely used for testing. Therefore, potentially pathogenic heterozygous mutations of TNFAIP3 may also be found in patients with no apparent HA20 phenotype.

There is no standard treatment for patients with HA20. Generally, treatment methods are based on the patient’s dominant clinical phenotype. The literature has reported that most patients respond to glucocorticoids, but the treatment cycle is often long-term. Interestingly, colchicine, which is traditionally used to treat familial Mediterranean fever, appears to have some effect on HA20, but has little effect in the treatment of other hereditary fever syndromes [22]. Slow-acting antirheumatic drugs such as methotrexate, cyclosporine, hydroxychloroquine, and mycophenolate mofetil have been reported in the literature, but most patients require a combination of glucocorticoids to control their symptoms. Because HA20 patients exhibit excessive production of pro-inflammatory cytokines, biologics targeting cytokines are commonly used as second-line treatments, and primarily include anti-TNF-α (infliximab, adalimumab), anti-IL-1 (anakinra, canakinumab), and anti-IL-6 (tocilizumab) [23], which can effectively inhibit systemic inflammatory responses in patients with HA20. Other immunotherapies, such as anti-CD20 monoclonal antibodies (rituximab) and JAK-1 and -3 inhibitors (tofacitinib) have also been reported. Hematopoietic stem cell transplantation can be considered for patients with severe refractory disease. A literature review of 24 cases showed 3 cases of effective treatment with colchicine, 1 case of ineffective treatment with colchicine but effective tocilizumab treatment, and 2 cases were initially treated with hormonal immunosuppressive agents followed by subcutaneous injection of 25 mg/w etanercept, which was effective, and 4 cases treated with steroid and immunosuppressive agents for disease control. Among our 3 patients, patient 1 experienced an allergic reaction after tow treatments with infliximab, thereby switched to adalimumab. At the same time, thalidomide was administered to control gastrointestinal symptoms. Patient 2 was treated with infliximab and sulfasalazine. Two patients showed significant clinical improvement after first application of TNF-α inhibitor. For Patient 1, her fever subsided, diarrhoea disappeared, and joint swelling and pain improved significantly. Lower back pain and joint mobility in patient 2 improved significantly. Considering the early age onset and serious side effects caused by steroid, steroid were not used in two of three patients. Follow-up at 4 months and 3 months showed disappeared joint swelling and pain (for who?). Patient 1 occasionally developed fever and bloody stool, but the frequency was significantly decreased. Most patients still required low-dose steroid therapy, in contrast to literature reports. Disease control using biologics and immunosuppressive agents in our three patients was satisfactory, but because of small number of cases and severity of the disease, treatment responses may differ. Therefore, more cases of TNFAIP3 mutation should be accumulated to elucidate the pathophysiology and treatment strategies for HA20 [24].

## Conclusions

A20 haploinsufficiency (HA20) is caused by a germline mutation with a highly penetrant loss of TNFAIP3 function. The discover of multiple nonsense mutations and frameshift mutations associated with impaired regulatory functions of TNFAIP3/A20 provides strong evidence that this is the pathogenic mutation. For patients with an early age onset, whole-exome genetic testing is necessary regardless of whether they present with characteristics of auto-inflammatory or auto-immune disease. For known rheumatic immune diseases of traditional significance, continuous improvement and widespread application of genetic testing technology have led to new diagnostic and therapeutic options for rheumatic immune diseases caused by single-gene mutations. Only early diagnosis and accurate treatment can result in better outcomes for patients.
